# Association of intervention outcomes with practice capacity for change: Subgroup analysis from a group randomized trial

**DOI:** 10.1186/1748-5908-3-25

**Published:** 2008-05-16

**Authors:** David Litaker, Mary Ruhe, Sharon Weyer, Kurt C Stange

**Affiliations:** 1 Department of Medicine, Louis Stokes Cleveland VA Medical Center, Cleveland, Ohio, USA; 2 Mannheim Institute for Public Health, Social and Preventive Medicine, University of Heidelberg, Germany; 3 Department of Family Medicine, Research Division, Case Western Reserve University, Cleveland, Ohio, USA; 4 Frances Payne Bolton School of Nursing, Case Western Reserve University, Cleveland, Ohio, USA; 5 Department of Sociology, Case Western Reserve University, Cleveland, Ohio, USA

## Abstract

**Background:**

The relationship between health care practices' capacity for change and the results and sustainability of interventions to improve health care delivery is unclear.

**Methods:**

In the setting of an intervention to increase preventive service delivery (PSD), we assessed practice capacity for change by rating motivation to change and instrumental ability to change on a one to four scale. After combining these ratings into a single score, random effects models tested its association with change in PSD rates from baseline to immediately after intervention completion and 12 months later.

**Results:**

Our measure of practices' capacity for change varied widely at baseline (range 2–8; mean 4.8 ± 1.6). Practices with greater capacity for change delivered preventive services to eligible patients at higher rates after completion of the intervention (2.7% per unit increase in the combined effort score, p < 0.001). This relationship persisted for 12 months after the intervention ended (3.1%, p < 0.001).

**Conclusion:**

Greater capacity for change is associated with a higher probability that a practice will attain and sustain desired outcomes. Future work to refine measures of this practice characteristic may be useful in planning and implementing interventions that result in sustained, evidence-based improvements in health care delivery.

## Background

Systematic reviews and meta-analyses demonstrate that many interventions to improve health care quality yield inconsistent results when evaluated through clinical trials [1,2]. One potential explanation for this is that the design of standardized interventions tends to overlook contextual factors that influence the implementation of new procedures in real world settings [3-8]. Exploring these factors further and testing their association with changes in health care delivery may therefore provide insights that foster more rapid uptake of evidence-based care into routine use.

Over the past two decades, work conducted in a broad range of settings has provided several ways to conceptualize influences on the implementation process [7,9-13]. One descriptive framework focusing on primary care practices' ability to adopt and implement new approaches to health care delivery[14] may be particularly valuable, given that these practices represent a venue through which a majority of Americans receive ambulatory care [15-18]. This framework, developed by Cohen et al., highlights the potential role of several practice characteristics: the individual and aggregate motivations of practice members; the resources that they identify within and outside the practice that are both accessible and important in supporting change efforts (including previous experience in using new tools or adopting new procedures); the external forces or factors that shape or influence change options; and practice members' perception of options and opportunities for change.

Two factors – motivations and resources for change – are central components of several frameworks for implementation[9,12,13], in addition to the one described by Cohen et al. While some have suggested that practice motivation or inertia may be important to clinical guideline implementations[19], motivation appears to be necessary but not sufficient for change to occur [9]. That is, confidence to act and an ability to implement change must also be present [9,12]. Other work lends support to this view: interventions that provide instrumental assistance during the implementation phase can be effective in fostering change once motivation exists or is developed [20]. Thus, it is important that studies examining the association of practice capacity for change with implementation use measures that represent both components and assess their potential interaction.

Despite considerable effort to characterize organizational capacity for change at the conceptual level, only a handful of studies have developed operational measures for this construct and established an association with implementation outcomes. Of these, the majority focus on intention to act rather than actual behaviors. To assess the association of capacity for change with demonstrable improvements in evidence-based health care delivery, we used data from the Study to Enhance Prevention by Understanding Practices (STEP-UP). This paper tests the hypotheses that greater practice capacity for change would be associated with greater change in the STEP-UP study outcome, preventive service delivery (PSD), from baseline to the end of the active intervention period and that these improvements would be sustained during follow up, when no intervention was being offered.

## Methods

The design, methods, and findings from STEP-UP have been described in detail previously [21,22]. In brief, this group randomized clinical trial to improve preventive service delivery randomly assigned 79 community-based primary care practices in northeast Ohio to a control or intervention group. Intervention practices were assessed by a research nurse facilitator over 1–3 days to gain an understanding of practice roles and routine procedures. The intervention, incorporating information from this assessment, involved creation of a practice-individualized plan for change using a menu of tools (e.g., chart stickers, flow sheets, reminder cards) and approaches (e.g., personnel roles, delivery of preventive care during illness visits) to enhance preventive health care. The study outcome, delivery of preventive services recommended by the U.S. Preventive Services Task Force[23], was determined through review of a cross-sectional sample of medical records for patients seen on a randomly selected day within two weeks of study baseline (month 0), month 6, month 12 (end of the intervention), and at follow up visits at months 18 and 24. PSD rates were calculated at each of these time points at each practice for each category of recommended services (e.g., screening, immunizations, and behavioral counseling). These three rates were then combined into a single global rate of PSD for each time point [24]. Thirty-nine practices were randomly assigned to the intervention; the 37 practices participating in follow up for the full 24 months represent the sample for this study.

Nearly all previous studies assessing organizational capacity use a quantitative approach that relies upon participant surveys [9,25-30]. While reflective of the experience or perspectives of those working in the practice, this approach is often limited by low response rates and may miss practice features that are not directly assessed by the items administered. To capture more fully the practice characteristics representing the conceptual domains of motivations and resources, we used a qualitative strategy based on direct observation of the practice by research team members. This process followed several steps. First, each member of the team (comprised of two nurse practice change facilitators, three research nurses involved in on-site medical record review, the epidemiologist data analyst and the physician/epidemiologist principal investigator) read extensive ethnographic field notes generated by the facilitator and research nurses from an assessment used to develop the practice-individualized intervention [20,31]. Each team member individually rated two aspects of the practice: the amount of effort needed to motivate practice staff to undertake the intervention (an inverse measure of the practice's a priori internal motivation to change), and the amount of instrumental assistance a practice needed to implement tools and approaches designed to increase PSD (an inverse measure of the practice's innate ability to change). Both ratings were expressed using a four-point scale. To facilitate model interpretation, scoring was reversed such that a value of one represented a practice in which substantial efforts were required to motivate the practice or to assist them in performing the instrumental tasks of the intervention (i.e., low intrinsic motivation or low ability to change); a value of four reflected the need for very little effort in either motivating the practice or in assisting it (i.e., high intrinsic motivation or high ability to change). The research team then met and shared their individual ratings. Discrepancies were resolved by discussing the practice from the diverse points of view of the team members. When necessary, original data were consulted to identify confirming or contradictory evidence for disparate ratings. As a final step, numeric ratings were added to form a single score representing the combined effort needed to motivate or to assist each practice in implementing the intervention.

To provide a preliminary test of the relationship between absolute change in PSD and the capacity for change score, we compared mean PSD values for practices in the highest and lowest tertiles of our score using Student's t-test. We then assessed this association more thoroughly using data from all study outcome assessments made every six months to develop models that accounted for repeated measures made at each practice. Separate models were developed to assess the association between the combined practice capacity for change score and change in the practice rate of PSD from baseline to month 12 and from baseline to month 24. A post-hoc analysis assessed the association of an interaction term (the product of both ratings) with the outcome at both time points. A two-tailed p value < 0.05 served as the threshold for statistical significance. SPSS version 13 and HLM version 6.03 were used to perform the analyses. The University Hospitals of Cleveland Institutional Review Board approved this study, which was conducted in accordance with the Declaration of Helsinki principles.

## Results

For the group as a whole, change in PSD from baseline to completion of the intervention period (month 12) varied significantly with absolute change ranging from -1% to 21% (mean 7.6% ± 5.5); at month 24, absolute change in PSD rates ranged from -9% to 26% (mean 6.9% ± 7.0). Regarding practices' capacity for change, the full range of scores (0–8) was used, with average score falling in the mid-range (mean 4.8 ± 1.6).

**Figure 1:**
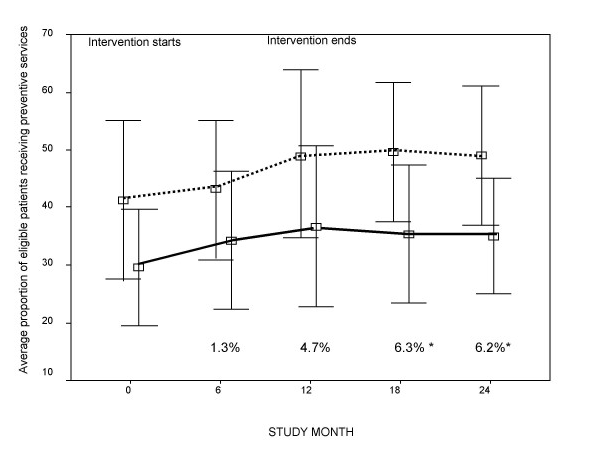
**Short title: Rates of Preventive Service Delivery at Practices with High and Low Capacity for Change**. Service delivery rates during and after an intervention to improve preventive care at a subset of practices estimated to have the highest and lowest capacity for change. Each box and bracket represent the mean and standard deviation, respectively, for PSD rates at assessments conducted every six months with mean difference in PSD rate adjusted for baseline presented at bottom. The ten practices with the highest capacity for change are indicated by the dashed line; the ten practices with lowest capacity for change are represented by the solid line. Note: PSD = preventive service delivery; *p < 0.01

We observed comparable rates of PSD improvement in the first 12 months for practices with both high and low capacity for change scores (Figure 1), with little difference in rates adjusted for baseline PSD. After month 12, however, significantly higher PSD rates were noted in the group of ten practices with the highest capacity for change. This finding was sustained through month 24, compared with the ten practices having lowest capacity for change (mean difference 6.2% ± 2.0; p = 0.009).

Using multiple assessments of outcomes at each intervention practice, random effects models demonstrated a 2.7% increment in PSD rates at month 12 (completion of the active intervention) for each unit increase in the practice capacity for change score (p < 0.001). This finding was similar at month 24 (3.1%; p < 0.001). To explore differences in PSD rates related to the components of the combined score, a supplemental analysis demonstrated a strong association with instrumental change capacity (3.2%, p = 0.002); a weaker association with motivation to change approached significance (2.1%, p = 0.09). A significant interaction was also observed: each one-point increase in the product of the two ratings (indicative of decreasing research team effort to motivate and to assist in instrumental tasks) was associated with an increase from baseline in PSD rates of 1.1% and 1.3% at months 12 and 24, respectively (both p values < 0.001).

## Discussion

These results yield two insights with potential value for implementation research. First, using qualitative estimates generated by our research team, we observe significant variation among practices in the level of effort required to motivate practices to undertake change and to assist them in implementing tools and approaches to enhance preventive service delivery. We also demonstrate that variation in our estimates of practices' capacity for change correlates with differences in outcomes both at the end of an intervention and for at least 12 months thereafter. Taken together, these findings suggest that these simple measures of capacity for change have utility in predicting intervention adoption, implementation, and maintenance and that variation in capacity for change may potentially explain inconsistent results of efficacious practice-based interventions applied outside controlled trial settings [5].

It is not surprising that variation exists in practices' capacity for change. Previous work, for example, highlights the rich differences that characterize the health system for primary care and the many factors that contribute to its evolution in individual practice settings [10,31-34]. Staff with particular skills, interests, and personal motivations, for example, enter and leave practices regularly, while new challenges within the larger health care system and in society continually emerge and dissipate [33]. Acknowledging these differences across practices may be useful for implementing efficacious interventions into real world practice settings in a variety of ways. In some cases, researchers seeking to enhance their success in improving health care delivery have begun to perform initial practice assessments and to use insights from this process to guide the development of tailored interventions [20,31,34-36]. An assessment of practice capacity for change may also be useful in promoting greater efficiency or equity in the deployment of an intervention, depending on the goals of the research team. Practice assessments, for example, may allow for the targeting of limited resources to practices with the greatest capacity for change. If resources are less limited, it may be possible to reduce practice-level disparities in performance by targeting greatest efforts toward those with the lowest capacity.

Given the nature of this analysis, we cannot establish a causal link between practice capacity for change and implementation outcomes. Our findings, however, provide justification for future replication studies as well as those that develop and test interventions to enhance both motivational and instrumental change capacity. Rationale for future developmental work in this area is further supported by evidence of a post hoc association between preventive service delivery and an interaction between these two factors sustained over time. Recent studies in commercial business settings now inform our understanding of ways in which motivation to change might be enhanced and new work patterns might be more readily adopted and implemented [37-40]. One strategy, for example, emphasizes organizational self-reflection to first identify and later leverage existing strengths (e.g., resources, personal motivations, and relationships) to build motivation within the group to undertake a project with shared meaning. In contrast to traditional quality improvement efforts, participants begin with a positive focus of what might be, rather than one that seeks to eliminate problems or to reduce gaps. Although its effectiveness in health care settings is currently under investigation, a recent report describes efforts to apply the self-reflective or appreciative approach to improve health care delivery in primary care [41]. Caution is advisable, however, in undertaking efforts to assess and modify motivations and abilities within a practice for the sake of greater implementation effectiveness, especially because the contribution of these features relative to that of other factors included in various conceptual models of organizational capacity for change is unclear. Previous comparative case studies of practices in STEP-UP show the possibility for surprises and missed opportunities, for example. Some practices undertake little change despite appearing to be highly motivated and capable of change, while others make large changes despite low capacity [20].

Our results should be interpreted within the context of several limitations. In the absence of a control group, we cannot exclude the possibility, for example, that unmeasured practice-level factors may have confounded the associations observed. Also, the sample of family medicine practices used may not have been representative of this diverse primary care specialty or of other primary care specialists (e.g., general internists, pediatricians) located elsewhere. Finally, we acknowledge that the qualitative estimates we used may have failed to capture important dimensions of practice capacity for change. Previous studies, for example, underscore the complexity of this construct [9,12-14]. Future work that develops these measures further is needed to enable a clearer understanding of the meaning and contribution of practice capacity for change to the adoption and routine delivery of evidence-based care.

## Conclusion

Greater practice capacity for change is associated with greater success in implementing and maintaining improvements in health care delivery. Efforts to acknowledge and address this practice characteristic may lead to greater intervention effectiveness and speed the dissemination of evidence-based care into community-based primary care settings.

## Competing interests

The authors declare that they have no competing interests.

## Authors' contributions

DL, MR and KS conceived of the study and participated in its design and coordination. All authors participated in the drafting of the manuscript and read and approved it in its final form.
